# Neuroablative central lateral thalamotomy for chronic neuropathic pain

**DOI:** 10.3389/fpain.2022.999891

**Published:** 2022-09-13

**Authors:** Anthony K. Allam, M. Benjamin Larkin, John P. McGinnis, Ashwin Viswanathan

**Affiliations:** ^1^ School of Medicine, Baylor College of Medicine, Houston, TX, United States; ^2^ Department of Neurosurgery, Baylor College of Medicine, Houston, TX, United States; ^3^ Department of Neurosurgery, University of Texas, MD Anderson, Houston, TX, United States

**Keywords:** posterior central lateral nucleus, thalamus, chronic neuropathic pain, ablative surgery, CLp, functional neurosurgery, cancer pain

## Abstract

Chronic neuropathic pain refractory to medical management can be debilitating and can seriously affect one's quality of life. The interest of ablative surgery for the treatment or palliation of chronic neuropathic pain, cancer-related or chemotherapy-induced, has grown. Numerous regions along the nociceptive pathways have been prominent targets including the various nuclei of the thalamus. Traditional targets include the medial pulvinar, central median, and posterior complex thalamic nuclei. However, there has been little research regarding the role of the central lateral nucleus. In this paper, we aim to summarize the anatomy, pathophysiology, and patient experiences of the central lateral thalamotomy.

## Introduction

Pain is often considered the 5th vital sign; however, even to this day, our treatment of chronic pain is still inadequate (1, 2). Chronic pain, defined as intractable pain lasting >3 months, is complex, difficult to treat and continues to affect a significant proportion of the global populations and remains the leading cause of disability worldwide (3). Neuropathic pain, a subtype of chronic pain, is often a result of somatosensory nerve dysfunction and typically is opioid non-responsive (4). Furthermore, a significant proportion of cancer patients experience chronic neuropathic pain that can become refractory to medical and surgical management, which greatly reduces their quality of life. Early surgical attempts at treating chronic pain aimed to disrupt the pathways that were responsible for the pathogenesis and transmission of pain through ablative procedures. Many regions of the brain were targeted including the thalamus, midbrain, and anterior cingulate cortex (5). However, due to numerous neurological and sensory complications that arose from surgeries at the pontine and mesencephalic level, stereotactic thalamotomies became the surgery of choice (5, 6). Early thalamotomies primarily targeted the lateral thalamus due to its role in relaying the sensory-discriminative aspect of pain; however, these interventions often resulted in high rates of somatosensory deficits (6–8). Medial thalamic targets had the benefit of lower rates of somatosensory deficits by instead retarding the affective-emotional aspect of chronic pain (7, 9–11). In recent years, there has been a resurgence in the use of ablative surgery (9, 12–14). However, even as the use of ablative surgery for chronic neuropathic pain has increased, there have been very few papers that have focused on the specific role that the central lateral thalamus plays in chronic neuropathic pain.

## Methods

**Figure 1:**
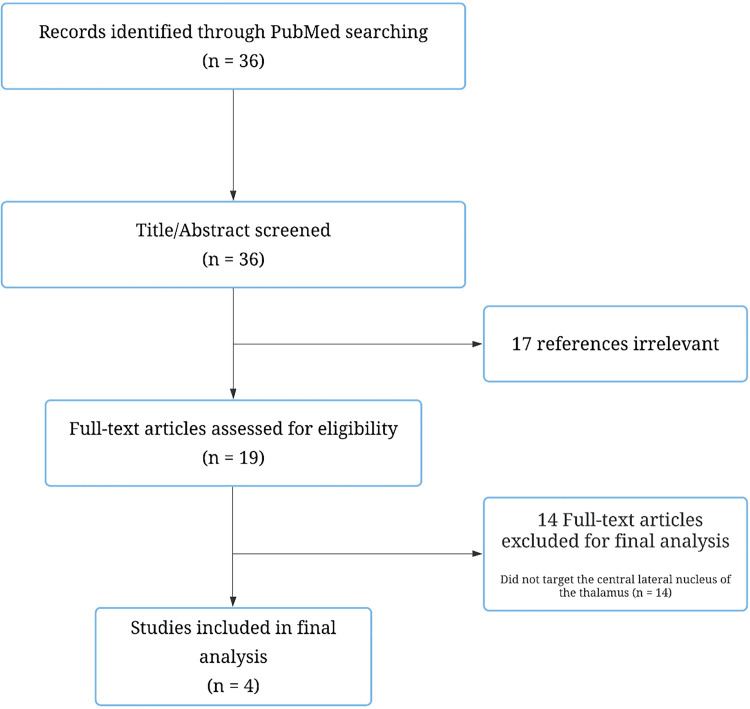
Flowchart depicting study selection process for final analysis.

PubMed was used as the primary database for electronic article searching. The National Library of Medicine's PICO (Patient/Population/Problem, Intervention, Comparison, and Outcome) guideline was used to guide the literature search terms. The terms pain, central lateral, centrolateral, medial, and thalamotomy were used. Only publications in English were considered. In addition, duplicate articles and articles with overlapping patient populations were accounted for to avoid redundancy. When articles with overlapping patient populations were seen, the study with the larger cohort of patients was used. This search yielded a total of 36 results which were then screened through an abstract and title review to yield a total of 19 papers. These 19 papers then underwent a full-text review by 2 authors (ML and AA) based upon the following criteria: (1) the study targeted the central lateral nucleus of the thalamus specifically, (2) the participants of the study had been suffering from pain for at least 6 months, (3) the study included post-treatment data, and (4) the study was written in English. Four studies that were used in the final analysis (Figure 1).

## Central lateral anatomy and physiology

It has long been understood that the lateral thalamus relays vital sensory and motor information from the periphery to cortical and sub-cortical domains. For example, the ventral posterior (VP) nucleus in the lateral thalamus receives information from the body and face through spinothalamic and trigeminothalamic tracts and relays them to the primary somatosensory cortex. However, ablative surgery in this region can result in somatosensory deficits due to the destruction of these pathways. In contrast, the medial thalamus represents a higher-order thalamic structure that receives few sensory inputs but regulates cognition, attention, memory processing, and reward-based behavior through cortico-thalamo-cortical pathways (15). As a result of its role in cognition, the medial thalamus affects the affective-emotional aspect of pain rather than the sensory-discriminative aspect seen in the lateral thalamus. In addition, lesions to the medial thalamus result in little to no somatosensory deficits as it spares the relay of sensory information to the primary somatosensory cortex. Previous targets have included the central median/parafascicular complex (CM/Pf), the posterior complex (POC), the central lateral nucleus (CL) and the medial pulvinar (PuM) (16–20). Within the medial thalamus, the posterior part of the central lateral thalamus (CLp) regulates the sensory, cognitive, and affective components of chronic neuropathic pain as it serves an intermediary role between the lateral and medial thalamic nuclei (5, 16).

The CLp is a key regulator of the thalamocortical (TC) network mediating afferent information from the spinothalamic and spino-reticulo-thalamic tracts and projecting to large areas of the cortex including the prefrontal cortex (PFC), posterior parietal cortex (PPC), the premotor and paralimbic (insula, ACC) areas; however, in the context of pain, the CLp becomes a dysfunctional regulator, perpetuating chronic neuropathic pain (16, 21–26). In a framework termed thalamocortical dysrhythmia, Jeanmonod proposed the transition of the normal CLp into a dysfunctional regulator as a result of the following steps (16, 21–25). Lesions either in the peripheral or central nervous system lead to the deafferentation of excitatory inputs to thalamic relay cells in the CLp in a process known as bottom-up deafferentation (16). In addition, deafferentation of thalamic relay cells can occur either through the top-down deafferentation from lesions within the cortical or sub-cortical domains (16). Or, the deafferentation of neighboring thalamocortical modules, identified as recurrent connections between the thalamus and higher-order cortical regions, by way of lesions within the thalamic VP nucleus (16). The loss of these excitatory inputs causes the hyperpolarization of cell membrane by activating T-type calcium channels that generate low threshold calcium spike (LTS) bursts in the 4 Hz range (16, 21, 27–29). These LTS bursts were first identified by Lenz et al. and Jeanmonod et al. later found these same LTS bursts to be spread diffusely in and around the CLp (16, 24, 26, 27, 30) These low threshold bursts, in the CLp, propagate throughout the thalamocortical module *via* the richly connected thalamocortical, thalamoreticular, corticothalamic, reticulothalamic, and cortico-reticulo-thalamic projections (21, 22). In turn, the affected thalamocortical module is also noted to have discharges at 4 Hz (16, 21, 22). The widespread projections result in neighboring areas to discharge at similar frequencies that results in continuous and widespread low frequency signals throughout the thalamocortical network (21, 22, 24). Furthermore, in cortical regions, reciprocal cortico-cortical inhibition is mediated by GABAergic interneurons. The widespread low frequency signals, in turn, limit the GABAergic inhibition of within these high frequency cortico-cortical connections (16, 21, 22). It is this increase in high frequency signals, especially in areas receptive to nociception, that is felt to produce the sensation of chronic pain.

**Figure 2:**
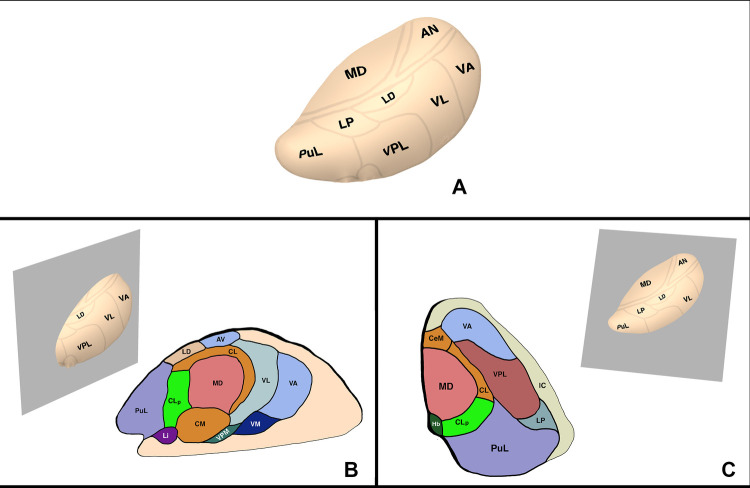
Thalamic anatomy. (A) External view. (**B**) Sagittal plane of the thalamus through the CLp. (**C**) Axial plane of the thalamus through the CLp. AV, anterior ventral nucleus; CM, centre median nucleus, CeM, central medial nucleus; CL, central lateral nucleus; CLp, posterior central lateral nucleus; Hb, habenular nucleus; LD, lateral dorsal nucleus; Li, limitans nucleus; LP, lateral posterior nucleus; MD, mediodorsal nucleus; PuL, pulvinar; VA, ventral anterior nucleus; VM, ventral medial nucleus; VPM, ventral posterior medial nucleus; VPL, ventral posterior lateral nucleus.

In addition to the physiological basis of the CLp as a target for chronic pain, the anatomical positioning of the CLp lends itself as a surgical target. In terms of anatomical location, the CLp is located 2 mm posterior to the posterior commissure (Anterior-Posterior) and 6 mm lateral to the thalamo-ventricular border (Medial-Lateral) on the AC/PC plane (16, 31). The low interindividual variability of the CLp especially in the anteroposterior axis makes the CLp an ideal surgical target. Small variations can be taken into account through MRI visualization of nearby structures such as the posterior commissure, the habenula, and the stria medullaris (5, 16, 31). In addition, damage to nearby structures such as the MD and PuM result in few neurological deficits and even a partial lesion of the CLp can provide enough de-amplification of the LTS bursts to provide clinical benefit (16) (Figure 2).

Furthermore, Jeanmonod reported that less than 1% of neurons in the CLp responded to sensory or motor information, indicating that the CLp had lost most of its regular function in patients with chronic pain (16, 27). This was especially true in patients who had chronic pain for over a year, where the likelihood of the CLp recovering to its previous level of function are low. However, the majority of normal CLp function can be taken over by other medial regulatory areas through thalamocortical plasticity. Therefore, surgical ablation of the CLp would leave the rest of the thalamocortical network unharmed, thereby reducing neurological and somatosensory complications.

Earlier researchers often targeted neighboring nuclei such as the CM/Pf, POC, and the PuM (13, 19, 32–34). However, patients who had lesions that encroached upon the CLp often had better results than those who did not (5, 12, 13, 17, 19). Stereotactic coordinates indicate that lesions of the more frequently researched nuclei are likely to encroach on the CLp.

Sano et al. targeted the posterior aspect of the medial thalamus; Hitchcock, Teixeira, and Young all utilized relatively large lesions in the posterior aspect of the central median/parafascicular (CM/Pf) complex, that invariably involved the CLp (8, 17, 19). This was again demonstrated by Urgosik and Lisack more recently who reported pain relief in 43% of their patients while targeting the posterior aspect of the CM/Pf complex (13).

Through an abundance of clinical, physiologic, and anatomical data, there is a strong *a priori* rationale for destructive lesioning of the CLp thalamic nucleus for the management of chronic pain. However, even as the use of ablative surgery for chronic neuropathic pain has increased, namely in alternative thalamic nuclei, there remains a dearth of information regarding the specific use of the CLp, which this paper intends to address.

## Central lateral thalamotomy

### Ablative modalities

Thalamic nuclei ablation has been done with a variety of techniques historically ranging from chemical agents to mechanical devices. Currently, the three main ablative techniques include: stereotactic radiosurgery (SRS), radiofrequency (RF) thermal ablation, and MR-guided focused ultrasound (MRgFUS) thermal ablation. These modalities are selected depending on the goal of the surgery, the patient's unique characteristics, and available equipment. The following paragraphs discuss specific studies utilizing one of the above ablative modalities and their respective benefits and limitations (Table 1).

**Table 1:** Studies of neuroablative CLp thalamotomy for chronic neuropathic pain.

Study, year	Frazini, 2021	Jeanmonod, 2001	Jeanmonod, 2012	Jeanmonod, 2020
Ablative modality	Gamma knife	RF thermal ablation	MR-guided focused ultrasound	MR-guided focused ultrasound
Patients included (included in outcome analysis)	8	96	12 (11)	8
Age of patients (years)	Mean (63.5)	Mean (56)	Range (45–75)	Mean (62)
Mean duration of chronic pain (years)	5.25	7.5	8.5	17
Pre-operative VAS score (out of 10)	9.4	8.5	5.95	8
3-Month mean pain relief (VAS Score)	20.2% (7.5)	-	42.4% (3.43)	85% (1.2)
1-Year mean pain Relief (VAS Score)	41.5% (5.5)	-	40.7% (3.53)	87.5% (1)
2-Year mean pain Relief (VAS Score)	52.1% (4.5)	-	-	-
Longest mean pain relief (VAS Score)	30.9% (6.5)	45.7% (4.62)	-	87.5% (1)

### Stereotactic radiosurgery

SRS delivers a large dose of radiation to the intended target causing cell death or halted mitosis (9, 35). There are multiple devices used to perform SRS with the two most common devices being Gamma Knife radiosurgery (GKRS) or linear accelerators. GKRS uses a beam of gamma rays created from the excited nucleus of cobalt, while a linear accelerator uses a high-energy beam of x-rays. Unlike RF thermal ablation, SRS can be delivered without operating on the brain, therefore reducing the risk of surgical complications. In addition, complex geometric shapes can be targeted using computerized programs that can deliver the radiation accordingly. However, unlike other ablative procedures, SRS has a delayed effect on creating the lesion as cell death takes place hours to days after the procedure is performed, and the clinical effect can in some cases take months to manifest. As a result, there is no intraoperative feedback, and the extent and shape of the lesion is more variable due to radiation fall-out effects.

In a study conducted by Franzini et al., 8 patients with neuropathic pain were treated either by a unilateral central lateral thalamotomy (CLT) or by a bilateral CLT by Gamma Knife SRS (maximal dose 130–140 Gy) (5). Four of the patients were afflicted with trigeminal deafferentation pain, 1 of them was afflicted with postherpetic neuralgia, another one was affected with central poststroke neuropathic pain, and the last 2 were affected with neuropathic pain secondary to a brachial plexus injury. Preoperative and postoperative pain and health outcomes were measured using four different surveys: Visual Analog Scale (VAS), McGill Pain Questionnaire (MPQ), EuroQol-5 dimensions (EQ-5D), and 36-Item Short Health Survey version 2 (SF-36v2). The mean age of the patients was 63.5 years with the mean duration of chronic pain before the procedure lasting 5.25 years. All patients had pain reduction following GKRS with the average time to initial pain reduction being 5.5 months and the median time to 50% pain reduction being 17 months. In terms of pain relief, 6 out of the 8 patients achieved >50% VAS pain score reduction with the average score dropping from 9.5 to 3.5 within 24 months of the follow-up period. Even within 1 year, the average VAS score significantly dropped from 9.4 to 5.5. In addition, there was improvement of the SF-36v2 quality of life survey with a mean increase of 48.16% and the EQ-5D survey with a mean increase of 45.16%. During the last follow-up, 5 out of the 8 patients reported >50% VAS reduction. There was also improvement for the MPQ (mean: −16.05%), the EQ-5D (mean: 35.48%), and SF-36v2 (mean: 35.84%) at the 24 month follow up. There were no lesion-related adverse effects that were seen in any of the patients. Pain did recur in one of the patients at the 6-month follow-up and in another patient at the 24-month follow up.

### Radiofrequency ablation

Radiofrequency thermal ablation creates a lesion *via* heat that is generated by an oscillating electrical field in the region of interest (36, 37). Intracranial electrodes are coupled to a RF generator that sends alternating electrical currents (at a frequency of 500,000 Hz for most modern machines) (9, 37). As a result, the ions in the nearby region oscillate as well producing friction and heat. Generally, RF thermal ablation results in distinct lesion borders that can be monitored intraoperatively; however, the size and shape of the lesions can be variable and there is a risk of surgical complications as the electrode is passed through the brain.

Jeanmonod et al. have published several studies on the treatment of chronic pain targeting the CL thalamus (16, 38). In the largest, they performed RF thermal ablation on 96 patients who had chronic neuropathic pain. The patients suffered from a variety of conditions with 58% having lesions in the primary afferents, 21% having a pure central lesion, and the remaining 21% having a mixed central plus peripheral lesion. It is important to keep in mind 5 patients in this study suffered from neuropathic pain secondary to cancer. The mean age of the patients was 56 years and mean time between pain onset and CLT was 7.5 ± 8 years. Pre- and post-operative pain assessments were measured using pain type, pain quality, VAS, and drug intake. Pain was further categorized into three different types: continuous (C), intermittent (I), and allodynic. The mean maximum pre-operative VAS was 85/100 ± 10. Overall pain relief resulted in a VAS 45.7 ± 39.6% with 53% of the patients estimating greater than 50% pain reduction, while 18% of the patients experienced complete pain relief. Further analysis of the data showed that intermittent pain was reduced to a greater extent and in more people than continuous pain perhaps indicating higher resistance of continuous pain to CLT. The mean pain relief estimated by patients with continuous pain alone or in combination with intermittent pain was 20.4 ± 25.8% in contrast to the mean pain relief of 66 ± 39.2% in patients with intermittent pain alone. In addition, thermal and proprioceptive qualities of allodynic pain were found to be more resistant to surgery. 31.6% of the patients had lower post-operative drug usage after the surgery. There were short-term complications in 10 patients during the first years of the study. These included intraventricular bleeding (*n* = 1), thalamic edema (*n* = 2), and partial and partially reversible pretectal deficits (*n* = 5). This was resolved in later years with no further complications following the use of different instrumentation, precise localization of the CLp, and an altered electrode route.

### MR-guided focused ultrasound

MRgFUS thermal ablation is a novel technique that creates a lesion using high intensity ultrasound beams (39). These beams are absorbed by the tissue in the target region and are converted to heat, causing tissue destruction (9, 39, 40). The ultrasound beams are created using a hemispheric phased array of transducers that are affixed to the skull. This allows the ultrasound beams to traverse the maximum available skull area to reach the target so as to avoid overheating and resultant brain damage. Some advantages of this technique include intraoperative monitoring, distinct lesion borders, and immediate results; however, this technique can only be used in central areas of the brain and the machinery causes claustrophobia in some patients.

The first use of MRgFUS for centrolateral thalamotomies was conducted on 12 patients with either central or peripheral causes for neuropathic pain (40). The patients ranged from 45 to 75 years old and had a mean duration of pain of 8.5 years. Three patients had inadequate lesions for the procedure, thus only the nine patients considered to have had the full treatment were included in the final results. These patients had lesions of 3–4 mm in diameter with mean peak temperatures at 53 ± 3.3°C. Thermocoagulative effects were seen from 50°C onwards with 100% necrosis being achieved around 55°C–57°C. Pre- and post-operative outcomes were measured using McGill Pain Questionnaire (MPQ), Visual Analog Scale (VAS), and global postoperative pain relief. The mean preoperative VAS score was 59.5/100 with the mean postoperative VAS score being 34.3/100 at 3 months and 35.3/100 at 1 year, a 42% and 41% improvement respectively. These patients experienced a mean pain relief of 49% at the 3-month follow-up and 57% at the 1-year follow-up. In addition, most of the patients had acute pain relief right after the surgery with a mean pain relief of 55% right after and 71.1% 2 days after. Six of the patients also had immediate and long-term somatosensory improvements. During the course of the study, there was one complication that resulted in ischemia of the motor thalamus secondary to a hemorrhage. In light of this incident, a cavitation detector was implemented, and the lesion temperatures were kept below 60°C for the remaining patients.

Another study conducted by Jeanmonod et al. focused on the use of MRI-guided focused ultrasound CLT albiet more specifically for trigeminal neuralgia (41). Eight patients with a mean age of 62 ± 12 years and a mean symptom duration of 17 ± 12 years were treated. Patients were either classified as idiopathic, classical, or secondary. Five of the patients had previous surgical interventions. Pre- and post-operative pain assessments were measured using McGill Pain Questionnaire (MPQ), Visual Analog Scale (VAS), and an estimation of pain relief by the patient. Final lesion temperatures were between 54°C and 58°C. The mean pain relief as stated by patients was 51% at 3 months, 71% at 1 year and 78% at the longest follow-up. This corresponds to pain reduction in 63% of patients at 3 months, 88% at 1 year and 100% at the longest follow-up. The mean preoperative VAS score measuring paroxysmal pain was 80/100. At the 3-month follow-up the mean VAS score dropped to 12/100, with the 1-year follow-up and longest-term follow ups having a mean VAS score of 10/100. There were no long-lasting adverse effects that were noted to be caused by MRgFUS of the CL thalamus.

## Discussion

This review highlights the physiologic, anatomical, and clinical basis for the use of various ablative techniques of the CLp thalamic nucleus and their respective outcomes for the treatment of chronic neuropathic pain not relieved with alternative medical or surgical interventions. A significant proportion of patients in the included studies benefited from CLp thalamotomy. The initial pain relief produced by the lesions is thought to be the result of the immediate cessation of low-frequency signals typically produced by the CLp and increased inhibition of GABAergic interneurons throughout thalamocortical modules that results in reduced high frequency signals within various cortical areas responsible for pain. But it is further known that the thalamocortical module has a tendency to resist change (21, 42). As the thalamocortical module slowly returns to normal, external factors like the patient's goals, the patient's attitude, and other social factors may also play a role in the resumption of normal thalamocortical activity (16).

In many of the included studies there were technical and procedural complications that could be attributed to operator technique and lack of experience within the respective ablative modality rather than unintended treatment effect. The complications seen in Jeanmonod et al. ablation using RFA were resolved in the later years of the study as a more precise localization of the CLp was uncovered along with the use of different instrumentation and electrode routes. Similarly, in his ablation of the CLp using MRgFUS, following the implementation of a cavitation detector and lower lesion temperatures, no further complications were seen.

Additionally, it is important to note that in none of the studies were there any long-term adverse effects that could be attributed to the lesion itself. There are likely two main reasons: first, that the CLT plays the role of a dysfunctional regulator in the setting of chronic pain and therefore it does not serve its normal role anymore. Many of the sensory and motor inputs from the spinothalamic and spino-reticulo-thalamic tracts no longer function in the context of chronic pain. Therefore, lesions to this area produce little to no somatosensory deficits. In addition, compared to other medial thalamic targets, the CLp is farther away from the lateral thalamus and particularly the ventral posterior region, thereby reducing the chance of iatrogenic injury and expected morbidity associated with these regions.

In many of the instances, patients achieved relatively long-term pain relief, with some achieving greater than 50% reduction in their pain even 2 years after the procedure. Comparing different surgical modalities for CLT, Gamma Knife has the longest onset until pain relief—an average of 5.5 months for initial pain relief and 17 months to achieve a 50% pain reduction. In cases when immediate pain relief is needed, other therapeutic options for CLTs are recommended. Furthermore, MR-Guided Focused Ultrasound provided similar or greater pain relief than the other modalities with fewer complications. SRS and MRgFUS, unlike RF ablation, can target the region of interest without disrupting neural structures along the planned trajectory.

It is important to recognize that three out of the four papers presented were published by the same author. Preliminary attempts resulted in more than desired complication rates. It is safe to say that the experience with the ablative target, CLp, and with the respective ablative modality is crucial for achieving maximum safety and clinical success and the above results should be considered appropriately.

Additionally, a prominent subset of patients who suffer from chronic neuropathic pain include cancer patients in which the prevalence is estimated to be as high as 40%. This pain can either arise from the disease burden itself, or as a result of various chemotherapeutic and radiosurgical treatments. Within this population, the pain can become opioid-resistant. This leaves patient with few remaining options for adequate pain control. In a review of patients who underwent SRS medial thalamotomies, 42% of whom had cancer-related pain, the majority of patient had meaningful pain reduction; and more posteriorly placed lesions were associated with better outcomes (7).

Within the above referenced trials, there was only a small number of patients who were underwent thalamotomy for cancer-related neuropathic pain. However, despite the lack of strong evidence in the form of randomized control trials and large sample sizes, the reader should remain optimistic that the presented results may translate to wider populations with continued investigation.

## Conclusion

There are multiple ablative modalities that target the central lateral thalamus for the treatment of chronic neuropathic pain. Current physiologic, anatomical, and clinical evidence consists of only a few small patient cohorts, many of which do not exclusively investigate cancer-related, or treatment-induced causes of pain. But it is our opinion that these provide reassuring support for continued investigation. Central lateral thalamotomies offers an effective, long-lasting pain target for ablative treatment of chronic neuropathic pain without the resultant neurological and somatosensory deficits commonly seen with other ablative targets. MR-Guided Focused Ultrasound may prove to be an invaluable tool in the neuro-ablative armamentarium for the treatment of refractory chronic neuropathic pain.

Given that each technique has its own strengths and criteria for use, it is ideal that we have numerous treatment options available. It is important to identify which treatment parameters and/or techniques, and in which patient populations, a CLT may provide patients with good long-term pain control. However, as this technique remains investigational, its use should continued to be monitored under the purview of an institutional review board. Additional, larger, randomized, adequately controlled trials focused on specifically cancer-related and chemotherapy induced causes for the management of refractory chronic neuropathic pain are necessary prior to any further more widespread therapeutic adoption.
